# Supplementary material for: Splice-Junction-Based Mapping of Alternative Isoforms in the Human Proteome
Source: Cell Rep. Author manuscript; Available in PMC 2020 Jan 15. (PMC6961840; doi:10.1016/j.celrep.2019.11.026)
Supplement: 2 [file NIHMS1546469-supplement-2.zip › DF1/PXD010154/Colon/Colon_13_ERI1_MHAGQLMSVSSSLPIEGTPPPQMPHFRNSTVAG.pdf]

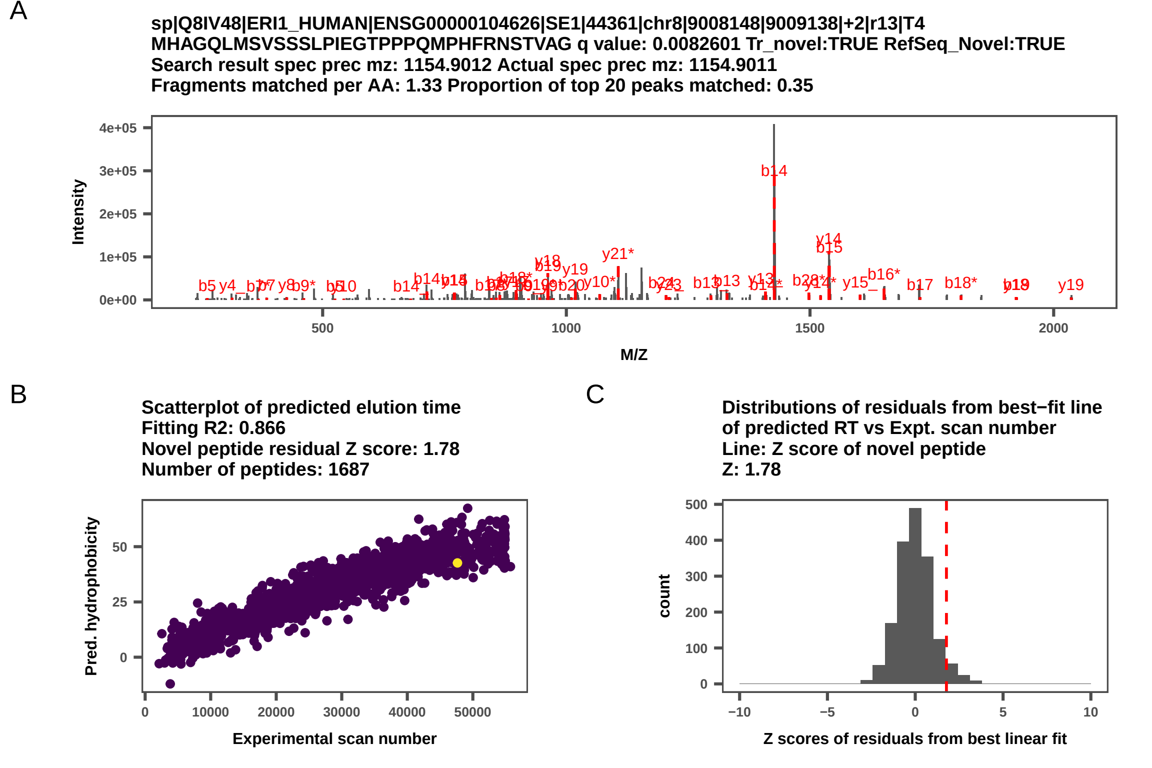
A
sp|Q8IV48|ERI1_HUMAN|ENSG00000104626|SE1|44361|chr8|9008148|9009138|+2|r13|T4
MHAGQLMSVSSSLPIEGTPPPQMPHFRNSTVAG q value: 0.0082601 Tr_novel:TRUE RefSeq_Novel:TRUE
Search result spec prec mz: 1154.9012 Actual spec prec mz: 1154.9011
Fragments matched per AA: 1.33 Proportion of top 20 peaks matched: 0.35
Intensity
4e+05
3e+05
2e+05
1e+05
0e+00
b14
y14
b15
y21*
y19
b16*
b17
b18*
y15
y19
b5
y4
500
1000
1500
2000
M/Z
B
Scatterplot of predicted elution time
Fitting R2: 0.866
Novel peptide residual Z score: 1.78
Number of peptides: 1687
Pred. hydrophobicity
50
25
0
0
10000
20000
30000
40000
50000
Experimental scan number
C
Distributions of residuals from best−fit line
of predicted RT vs Expt. scan number
Line: Z score of novel peptide
Z: 1.78
count
500
400
300
200
100
0
−10
−5
0
5
10
Z scores of residuals from best linear fit
